# Optic neuritis of MOG-IgG-associated autoimmune disorders: a case report

**DOI:** 10.1186/s12886-020-01780-8

**Published:** 2021-01-09

**Authors:** Tiantian Li, Jian Zhou, Xiaoling Yan, Ran Duan, Xiaobo Zhu

**Affiliations:** 1 Beijing University of Chinese Medicine, No.11 Bei San Huan Dong Lu, Chaoyang District, Beijing, 100029 China; 2 Ophthalmology Department, Beijing University of Chinese Medicine Affiliated Dongfang Hospital, No.6 Fangxingyuan 1st Block, Fangzhuang, Fengtai District, Beijing, 100078 China; 3 Medical Affairs Department, Beijing University of Chinese Medicine Affiliated Dongfang Hospital, No.6 Fangxingyuan 1st Block, Fangzhuang, Fengtai District, Beijing, 100078 China

**Keywords:** Case report, Myelin oligodendrocyte glycoprotein (MOG) antibodies, Antibody testing, Optic neuritis (ON), Neuromyelitis optica spectrum disorders (NMOSD), Multiple sclerosis (MS)

## Abstract

**Background:**

The diagnosis of immunoglobulin G serum antibodies to myelin oligodendrocyte glycoprotein (MOG-IgG) associated inflammatory demyelinating disorders can be confirmed by the presence of MOG-IgG, yet its general cut-off concentration had not yet to be defined. Whether it is significant that a seropositive lower titer level for MOG-IgG could cause disease is still unknown.

**Case presentation:**

A 55-year-old Chinese woman presented with acute optic neuritis manifestations in the left eye. MRI showed a left optic nerve demyelination image and a T2 hyperintensity at C7 vertebral segment without any extra specific lesions. AQP4-IgG was tested seronegative, while the MOG-IgG was positive, titer 1:10, by indirect immunofluorescence. Considering the lower concentration, we retested serum MOG-IgG after 6 months of steroid therapy, using cell-based assay, then we still got the same result which was also barely above the negative cut-off value. So, the clinical diagnose was “possible MOG-IgG-associated encephalomyelitis”. The woman’s condition improved by steroid therapy without relapse.

**Conclusions:**

Seropositive MOG-IgG, even at a lower level, could lead to an autoimmune inflammatory demyelination. In adults, it commonly presents as ON and myelitis. Although the patient had a considerable reaction, steroid therapy could not make MOG-IgG seronegative, instead, the antibody may persist even during remission and flare-ups can recur after steroid withdrawal. Therefore, a long-term follow-up is necessary to monitor the patient’s prognosis.

## Background

Myelin oligodendrocyte glycoprotein (MOG) is a protein on the outermost layer of myelin sheath in central nervous system (CNS) [1]. As a candidate of CNS autoantigen, however, MOG is considered to be an autoantibody (MOG-IgG) target for T- and B-cell responses. In recent studies, a new-generation cell-based assay (CBA) have demonstrated an association of MOG-IgG with inflammatory CNS demyelinating disorders, like acute demyelinating encephalomyelitis (ADEM), optic neuritis (ON) and myelitis [2]. Although it is detected that median MOG-IgG serum titers were significantly higher during an acute attack or a relapse course [3], the general cut-off value for MOG-IgG had not yet to be defined. We reported a woman who primarily attacked by severe ON with MOG-IgG seropositive at a lower titer level. According to the international recommendations of MOG-IgG-associated encephalomyelitis (MOG-EM) (published in 2018) [4], we decided to make a diagnosis of “possible MOG-EM”.

## Case presentation

**Fig. 1:**
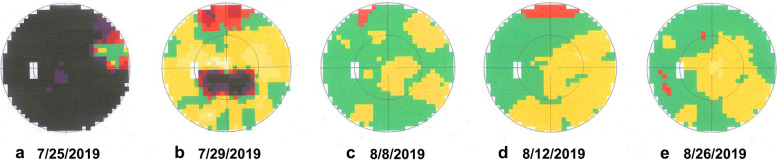
Perimetrical Test. **a**. only a small residual view remained on nasal quadrant; **b**. a remaining central scotoma; **c**-**e**. improved visual field

**Fig. 2:**
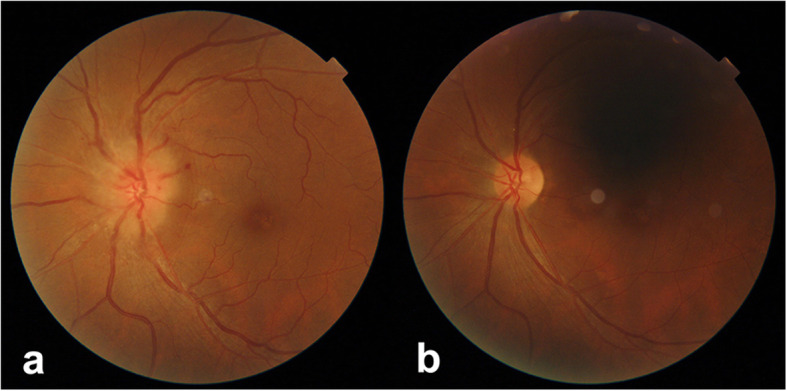
Fundus photography. **a**. diffuse disc edema and vascular angiectasis with linear hemorrhage around optic disc. **b**. the border of disc was clear without hemorrhage; the retinal arteries and veins were roughly normal

**Fig. 3:**
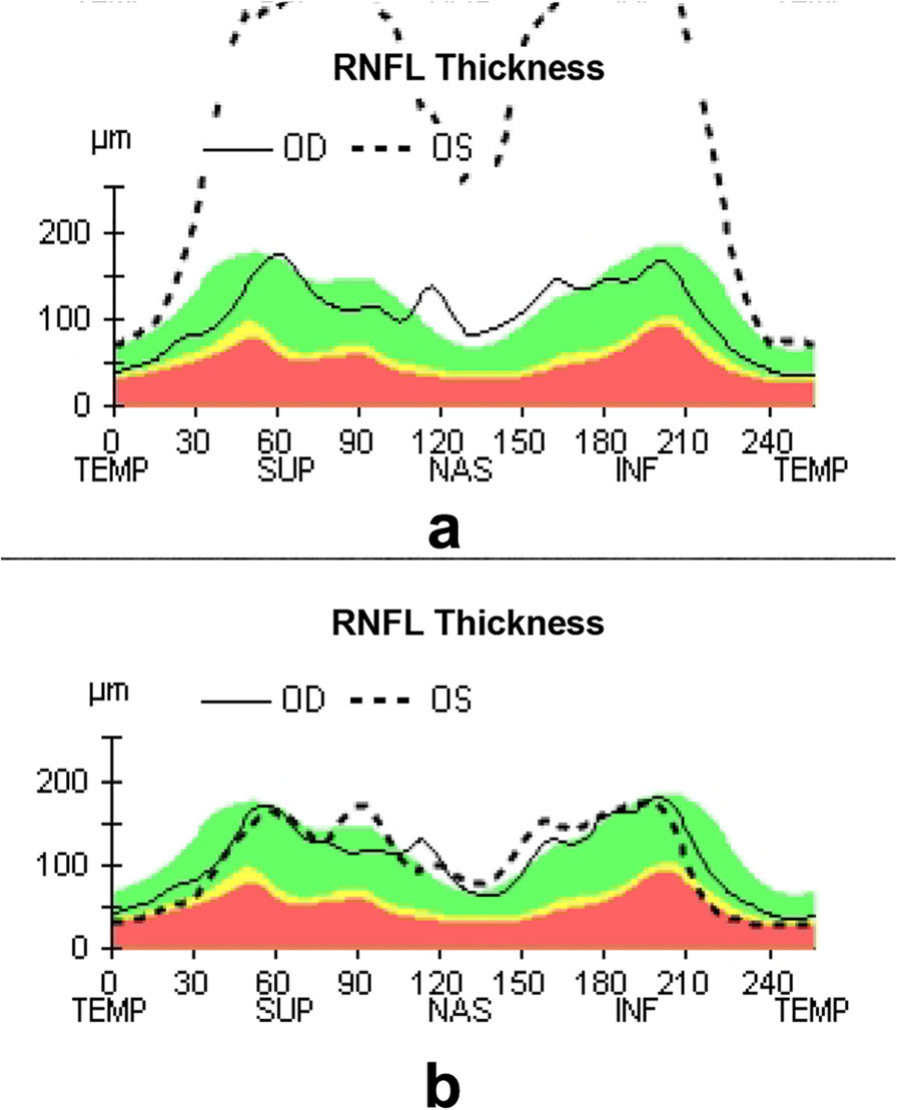
optical coherence tomography, OCT. **a**. Thickening in all RNFL quadrants and the average RNFL thickness rose dramatically to 347 μm at first visit. **b**. A month later, the RNFL thickness decreased nearly to normal level. The average RNFL thickness recovered to 106 μm

**Fig. 4:**
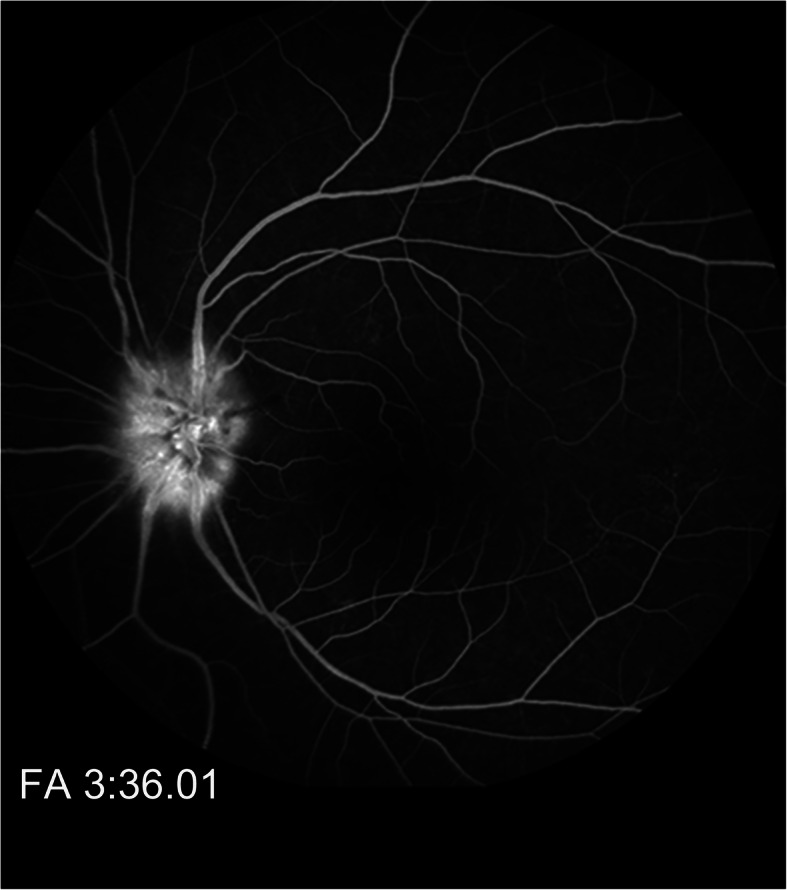
fundus fluorescein angiography, FFA. Diffuse high fluorescence leakage and linear low fluorescence in the left optic disc

A 55-year-old woman presented with decreased visual acuity (VA) in the left eye accompanied by periocular pain lasting for 2 weeks. She caught a cold 5 days before the ophthalmological symptoms set on. Later, the VA of the left eye decreased to 0.4 (logarithmic visual acuity chart) and an edematous optic disc was found on ophthalmoscopy. Although she was treated by Pred Forte Eye Drop for 5 days, followed by retrobulbar injection of Racanisodamine Hydrochloride, the VA continued to decline. Her past medical history included 15-years hypertension and lumbar decompression in 2002. The VA of the left eye was couting finger at 15 cm with relative afferent pupillary defect, while the VA of the right eye remained 1.0. Perimetrical Test showed only small residual view remained in the nasal quadrant (Fig. 1.a). Diffuse disc swelling and vascular angiectasis with linear hemorrhage around optic disc were captured on Fundus photography (Fig. 2.a). The average peripapillary retinal nerve fiber layer (RNFL) thickness of left eye increased to 347 μm (Fig. 3.a). Fundus fluorescein angiography reminded diffuse high fluorescence leakage and linear low fluorescence in left optic disc (Fig. 4). Flash visual evoked potential (F-VEP) showed P-wave, at 1.0 Hz, prolonged (130.6 ms) and electric voltage decreased (8.96 μV). Neurological examination showed normal muscle strength in all extremities, no sensory deficits, normal deep tendon reflexes, and no signs of bladder nor bowel dysfunction. Furthermore, brain and spine MRI captured a corresponding optic nerve demyelination image with no involvement of optic chiasm and a T2 hyperintensity only at C7 vertebral segment without any extra specific lesions. Serum TORCH test showed rubella virus IgG of 59.9 IU·ml^− 1^ (neg: < 10), cytomegalovirus IgG 425.5 IU·ml^− 1^ (neg:< 0.5), herpes simplex virus IgG 1.19 IU·ml^− 1^ (neg: < 0.6). T-cell indexes revealed the active CD4+ (1230/μl, reference value was 550–1200/μl) and CD8+ (1037/μl, reference value was 380–790/μl), while autoimmune screening including anti-nuclear antibodies, complement levels, thyroid-relevant antibodies and rheumatoid factors, was atypical. However, aquaporin-4 (AQP4)-IgG was seronegative assayed by ELISA, while the indirect immunofluorescence (IIFT) demonstrated that MOG-IgG was identified in the serum (titers: 1:10). Given all that, the clinical diagnose we considered was “possible MOG-EM”.

To relief her symptoms, three courses of intravenous methylprednisolone (IVMP) (1000 mg·d-1, for 3 days, and halved every 3 days) were prescribed, followed by oral administration of 60 mg·d^− 1^ prednisolone. Oral dose was reduced gradually to 24 mg in maintenance, as she responded well to the treatment (changes in view field, optic disc formation and RNFL thickness showed by Fig. 1.b-e, Fig. 2.b and Fig. 3.b respectively). A month later, her visual acuity was improved to 0.6 for the left eye. The intervention adherence of this patient was good, with an average review every week. After six-months follow-up, there was no recurrence and any adverses or unanticipated events happened. Re-tested by a new CBA method, her MOG-IgG titer was still 1:10. Now, one tablet of prednisolone (5 mg) was taken per day to stable symptoms at the last follow-up.

## Discussion and conclusions

This patient once was misdiagnosed as AQP4-IgG-negative neuromyelitis optica spectrum disorders (NMOSD) because of a positive serological result for MOG-IgG, while lack of AQP4-IgG. However, the patient presented with isolated ON, and lacked dissemination in space; brain MRI only showed the corresponding demyelinating lesions in left optic nerve without optic chiasm, and no extra specific lesions were reported in the cerebrum; MRI of the spinal cord only reported an isolated short segment lesion instead of three or more vertebral segments. According to the international consensus diagnostic criteria for NMOSD, revised by international panel for NMO diagnosis (IPND) in 2015 [5], the clinical and imaging manifestations in this case do not actually support the diagnosis of AQP4-IgG-negative NMOSD. Excepting the common clinical presentations, such as acute decrease of visual acuity, periocular pain, and visual field deficits, it is worth noting that this patient’s swollen optic disc showed linear retinal hemorrhage, but AQP4 related NMOSD rarely manifests as optic disc bleeding [6]. In addition, it is reported that similar as AQP4-IgG-positive NMOSD, AQP4-IgG-negative NMOSD often involves cervical-thoracic spinal cord lesions, while MOG-IgG-positive MRI often involves lower spinal cord segments lesions as well as the conus lesion [6]. The corresponding sphincter and erectile disturbance may occur concurrently with acute CNS injury. Unfortunately, with no obvious clinical manifestations, the patient rejected the spinal cord MRI examination of the lumbosacral region, so we could not obtain relevant reference evidence.

According to international recommendations of MOG encephalomyelitis (2018), the clinical, imaging presentations of acute optic neuritis, MOG-IgG seropositive, and the evidence of T cell activation, all met the diagnostic criteria of MOG-EM [3, 4]. However, the recommendations also mentioned some atypical “red flags” which challenged the MOG-EM diagnose. Thus, several laboratory results of this patient really need to be vigilant. First, MOG-IgG titers was barely above the assay-specific cut-off, and the clinical picture on brain MRI was atypical. Second, rubella and herpes simplex virus were positive in TORCH test, in which condition was suggested that multiple sclerosis (MS) should be considered. However, In comparison with the MS criteria (2017) [7], Dawson’s finger-type or round/oval or juxtacortical U fiber lesion found on brain MRI of MS patients, are lacked in typical MOG-IgG-positive patients; cerebrospinal fluid (CSF)-specific oligoclonal bands, indicating a diagnosis of MS, is also absent in MOG-IgG-positive patients; moreover, MOG-IgG itself is extremely rare in adults with MS. Thus, the evidences in this case does not fulfill the MS diagnosis criteria. After about 6 months of oral steroid therapy, there was no increase or decrease in the re-tested MOG-IgG titer level (still 1:10), assayed by another CBA method. By comprehensive analysis, the diagnosis as “possible MOG-EM”, suggested in the recommendations, was given.

A retrospective study of 50 MOG-IgG-positive patients suggested that adolescent had higher serum antibody titers than adults, and ADEM was mainly associated with young children, while presentations with ON or myelitis was common in older children and adults [8]. However, there is no general reference cut-off of pathological changes caused by MOG-IgG currently. It just emphasized that MOG-IgG serum titers were depend on disease activity, significantly higher median titers during acute attacks than during remission, and treatments status [4]. However, at the time of first onset of this patient, the concentration of MOG-IgG was at a low level while the acute ON manifestations were obvious. After IVMP and 6 months of oral steroid hormones to stable symptoms, the result of serum antibody titers did not reduce, indicating that lower concentration of serum MOG-IgG may also cause the central demyelination in the elderly cohort.

Compared with AQP4-IgG-positive NMOSD and classical MS, the prognosis of MOG-EM is optimism [9, 10]. So low-dose oral steroids were still prescribed for this patient to prevent recurrence. But considering that MOG-IgG persists in the CNS, the possibility of flare-ups after steroid withdrawal could not be ruled out. It was reported that the relapse of MOG-EM will occur within 9–12 months after the treatment [8]. Therefore, this patient still needs a long-term follow-up observation.

## Data Availability

All data generated or analysed during this study are included in this published article and its supplementary information files.

## Abbreviations

AQP4: Antiaquaporin-4
ADEM: Acute demyelinating encephalomyelitis
CBA: Cell-based assay
CNS: Central nervous system
CSF: Cerebrospinal fluid
F-VEP: Flare visual evoked potential
IIFT: Indirect immunofluorescence
IPND: International panel for NMO diagnosis
IVMP: Intravenous methylprednisolone
MOG-EM: MOG-IgG-associated encephalomyelitis
MS: Multiple sclerosis
MOG: Myelin oligodendrocyte glycoprotein
NMOSD: Neuromyelitis optica spectrum disorders
ON: Optic neuritis
RNFL: Retinal nerve fiber layer
VA: Visual acuity

## References

[1] Ramanathan S, Dale RC, Brilot F (2016). Anti-MOG antibody: the history, clinical phenotype, and pathogenicity of a serum biomarker for demyelination. Autoimmun Rev.

[2] Cobo-Calvo Á, Ruiz A, D'Indy H (2017). MOG antibody-related disorders: common features and uncommon presentations. J Neurol.

[3] Jarius S, Ruprecht K, Kleiter I (2016). MOG-IgG in NMO and related disorders: a multicenter study of 50 patients. Part 1: Frequency, syndrome specificity, influence of disease activity, long-term course, association with AQP4-IgG, and origin. J Neuroinflammation.

[4] Jarius S, Paul F, Aktas O (2018). MOG encephalomyelitis: international recommendations on diagnosis and antibody testing. J Neuroinflammation.

[5] Wingerchuk DM, Banwell B, Bennett JL (2015). International consensus diagnostic criteria for neuromyelitis optica spectrum disorders. Neurology..

[6] Jarius S, Wildemann B (2019). Devic's index case: a critical reappraisal - AQP4-IgG-mediated neuromyelitis optica spectrum disorder, or rather MOG encephalomyelitis?. J Neurol Sci.

[7] Carroll WM (2018). 2017 McDonald MS diagnostic criteria: evidence-based revisions. Mult Scler.

[8] Jarius S, Ruprecht K, Kleiter I (2016). MOG-IgG in NMO and related disorders: a multicenter study of 50 patients. Part 2: Epidemiology, clinical presentation, radiological and laboratory features, treatment responses, and long-term outcome. J Neuroinflammation.

[9] Höftberger R, Sepulveda M, Armangue T (2015). Antibodies to MOG and AQP4 in adults with neuromyelitis optica and suspected limited forms of the disease. Mult Scler.

[10] Traboulsee A, Simon JH, Stone L (2016). Revised recommendations of the consortium of MS centers task force for a standardized MRI protocol and clinical guidelines for the diagnosis and follow-up of multiple sclerosis. AJNR Am J Neuroradiol.

